# Was severe SARS-CoV-2 substantially spreading in Northern Italy before its first detection in February 2020? An evaluation of pneumonia-associated hospitalization trends from September 2014 to February 2020

**DOI:** 10.1093/eurpub/ckaf137

**Published:** 2025-08-04

**Authors:** Elisa Di Maggio, Daniele Petrone, Martina Del Manso, Flavia Riccardo, Antonino Bella, Silvio Brusaferro, Patrizio Pezzotti

**Affiliations:** Hygiene Unit, Department of Medical and Surgical Sciences, University of Foggia, Foggia, Italy; Department of Infectious Diseases, Istituto Superiore di Sanità, Rome, Italy; Department of Infectious Diseases, Istituto Superiore di Sanità, Rome, Italy; Department of Statistics, Sapienza University of Rome, Rome, Italy; Department of Infectious Diseases, Istituto Superiore di Sanità, Rome, Italy; Department of Infectious Diseases, Istituto Superiore di Sanità, Rome, Italy; Department of Infectious Diseases, Istituto Superiore di Sanità, Rome, Italy; Department of Medical Area, University of Udine, Udine, Italy; Department of Infectious Diseases, Istituto Superiore di Sanità, Rome, Italy

## Abstract

Retrospective studies identified SARS-CoV-2 worldwide circulation as early as late 2019. In Italy, the first autochthonous COVID-19 case was diagnosed in a Northern Region on 20 February 2020, raising the question whether high numbers of COVID-19 pneumonia cases were previously undetected. We explored whether unusual increases in hospitalizations for pneumonia occurred from October 2019 to February 2020 in Italy, particularly in Northern Regions. We analysed the Italian National Hospital Discharge Records with pneumonia ICD-9-CM codes from 2014 to 2020. Trend analysis and generalized linear models with negative binomial distribution were applied to compare observed pneumonia trends in the study period with previous years. Analyses were stratified by major regions (NUTS1) and provinces. During the study period, 2 501 074 hospitalizations were coded as pneumonia. No unusual increases of all hospitalizations associated to pneumonia were observed until mid-February 2020. Hospitalizations with viral pneumonia ICD9-CM codes were negligible until the end of January 2020, with a significant increase in two provinces of Lombardy Region 1–2 weeks before the first autochthonous COVID-19 case. Our analysis showed that a small increase in viral pneumonia hospitalizations in Northern Italy only in the weeks immediately preceding the first locally acquired SARS-CoV-2 case in two provinces of Lombardy. This excludes large-scale circulation in the last months of 2019 and in January 2020. Given the mild 2019–2020 influenza season and lower pneumonia hospitalization burden, the initial increase could have been interpreted as a fluctuation as it did not determine an overall excess case-load of pneumonia hospitalizations.

## Introduction

In December 2019, China confirmed the first cluster of patients with a novel severe acute respiratory syndrome due to a new coronavirus that would be late known as SARS-CoV-2 [1]. From 27 January 2020, the European Centre for Disease Prevention and Control (ECDC) and the World Health Organization (WHO) Regional Office for Europe launched standard case reporting of severe acute respiratory infection (SARI) cases with travel history to affected areas, with contextual conditions (being a health care worker or an unusual/unexpected clinical deterioration) and with evidence of possible SARS-CoV-2 exposure (e.g. direct contact with a case, exposure in an affected healthcare context or contact with animal hosts—if identified—in affected areas) [2]. Following the declaration of a Public Health Emergency of International Concern by WHO [3], on 31 January 2020 a state of national emergency was also declared in Italy with the immediate strengthening of surveillance measures to prevent, contain and mitigate the spread of this infection. The existing SARI surveillance system was reinforced in late January by testing patients also for COVID-19 following indications from Ministerial Circular no. 1997 [4], and in line with subsequent WHO recommendations [5]. In the earliest stages of the pandemic, this system gathered data on cases of SARI which could meet the international case definition for new coronavirus infection. In late January 2020 this surveillance system detected the first cases of suspected SARS-CoV-2 infection among Italian and foreign citizens with travel-history to China. After the detection of the first locally acquired case of infection on 20 February 2020, epidemiological investigations were enhanced and the number of locally acquired laboratory-confirmed cases increased rapidly, initially in Northern Italy and subsequently throughout the country [6] with an estimated basic reproductive number (*R*_0_) between 2.5 and 3 [7]. By 8 March 2020, nearly 10 000 COVID-19 hospitalizations and over 10 500 new diagnoses were reported to the national COVID-19 integrated surveillance system [7] (Supplementary Fig. S1). The majority of initial cases and hospitalizations occurred in the Lombardy region, in Northern Italy, which accounted for ∼71.8% of hospitalizations and 58.6% of diagnoses, with substantial pressure on the healthcare system. The rapid global spread of COVID-19 led the WHO to declare it a pandemic on 11 March 2020 [8].

Retrospective analyses of clinical samples [9–11] and environmental studies on wastewater surveillance [12, 13] indicated in later years that in the last months of 2019 the virus was already circulating in many European countries, including Italy. For this reason, in reconstructing the early pandemic response in Italy, many wondered whether a relevant number of undiagnosed hospitalizations due to COVID-19-pneumonia might have occurred before February 2020 and have been missed [14]. This has led to an ongoing debate on the public health authorities’ inability to identify timely signals of an abnormally high number of severe respiratory illnesses, between the end of 2019 and the beginning of 2020, which could have helped to implement earlier containment measures and potentially mitigate the impact of the pandemic [15, 16].

With this study, we aimed to retrospectively assess whether detectable signals of an increase, compared with previous years, in the number of hospitalizations with pneumonia were present in the months preceding the detection of the first locally acquired infection. We retrospectively analysed pneumonia hospitalization trends in Northern Italy from the beginning of the 2014/15 influenza season to the start of the national lockdown on 8 March 2020.

## Methods

### Study design and data sources

We conducted a retrospective analysis using anonymized individual data extracted from the Italian National Hospital Discharge Registry (HDR) in the period 2014–20. This registry contains health care data on all hospitalizations that have occurred throughout the country by collecting hospital discharge forms (HDFs) [17] which include personal data, hospital permanence (i.e. type of discharge, admission and discharge dates, length of stay), and clinical information (i.e. principal and secondary diagnoses, diagnostic, or therapeutic procedures) [18]. Clinical information and diagnostic procedures are coded according to the ‘International Classification of Diseases, 9th Revision, Clinical Modification’ (ICD-9-CM) [19]. We defined hospitalizations with pneumonia those with the ICD-9-CM codes reported in Supplementary Table S1. All hospitalizations were selected when at least one of these codes was reported as principal or secondary diagnosis. Although the national HRDs include both hospitalizations and day hospitals, the latter have been excluded from the present study because it refers to a daily admission or to a planned cycle of daily admissions lasting less than 12 hours and does not include situations where patients are hospitalized for acute, unplanned illnesses. Each admission was considered as a distinct event, despite the possibility of multiple hospitalizations per patient.

### Statistical analysis

Data presented here refer to the date of admission in the hospital. Descriptive and time series analysis techniques were used to assess if there were signals related to pandemic emergence before the first autochthonous case was laboratory confirmed (20 February 2020). The analyses here presented mainly refer mainly to Northern Italy because it was the territory most impacted in the first COVID-19 epidemic, particularly the Region of Lombardy [7].

We considered weekly hospitalizations with pneumonia (from Monday to Sunday), from 29 September 2014–8 March 2020, taking into account the first Monday of the 2014/15 flu season and the last Sunday before the national lockdown [20, 21]. We first examined the Northern Italy trend of admissions with pneumonia diagnosis. Following this, we provided a more detailed analysis of Lombardy and its provinces, from 1 October 2018, to examine the trend of admissions from the 2018/19 influenza season to the conclusion of the study [22]. We then examined the trend of pneumonia hospitalizations in each Region/autonomous province (AP) in the same period and provided also trend for Central-Southern Italy and the whole country. Northern Italy is geographically defined as the union of the following regions/ AP: Valle D’Aosta, Piedmont, Lombardy, Bolzano AP, Trento AP, Veneto, Friuli Venezia Giulia, and Emilia Romagna, NUTS2 according to Eurostat [23].

To better understand if the observed cases of pneumonia from week 39-2019 to week 9-2020 were in line with the expected trend, we applied negative binomial multivariable models with fixed effects to data from week 39 of 2014 to week 38 of 2019. The results of these models were then used to predict expected cases in the subsequent period. These models were better suited than the Poisson models, most commonly used for counts data, because, due to overdispersion, the basic assumption that mean and variance parameters are equal, was not respected. The models included as covariates week-date (trend component), sex, weekly incidence of influenza like illness (ILI) (×1000 assisted) [24], age group (5-year age group from 0–4 to 95–99, ≥100), position of the pneumonia code on the HDR (principal or secondary cause of admission), period of school openings [25] (coded as 1 up to 8 weeks from the national opening day in September, 0 otherwise), annual seasonal components [estimated by sin (2*π*week/52) and cos (2*π*week/52)], and the interaction among week-date and seasonal components. Weekly ILI incidence was retrieved from National Health Service Sentinel System (InfluNet) [21, 26]. Since ILI’s surveillance in Italy was active only during the winter season (from week 42 to week 17 of the following year), we estimated the ILI incidence for the weeks when surveillance was inactive using a second-degree loess curve. This curve takes into account the last two data points observed from the previous winter season and the first two data points observed from the following winter season.

We then calculated the difference between the number of observed hospital admissions with pneumonia and their predicted number from 30 September 2019–8 March 2020. To obtain 95% confidence intervals for the estimated values, we used the bootstrap method with 1000 repetitions extracting the 2.5 and the 97.5 percentiles of the distribution obtained. We also ran models using data from the whole country, from the Lombardy Region, and the two most affected provinces, Bergamo and Lodi [27]. Finally, we restricted this analysis to individuals hospitalized with only viral pneumonia (ICD-9-CM code 480) in Lombardy Region, Bergamo, and Lodi province. Due to the low number of viral pneumonia cases in the province of Lodi, which prevented model convergence, we included as covariate only week date, annual seasonal components and the interaction among them.

Furthermore, to provide a comparison with data coming from other data sources, we retrospectively, analysed the trend of negative results on persons with ILI and tested molecularly for influenza in Lombardy Region during the 2017/2018, 2018/2019, and 2019/2020 seasons [28].

All the analyses and the figures were carried out with RStudio 2023.12.1 under R 4.3.2 [29].

## Results

**Figure 1.**
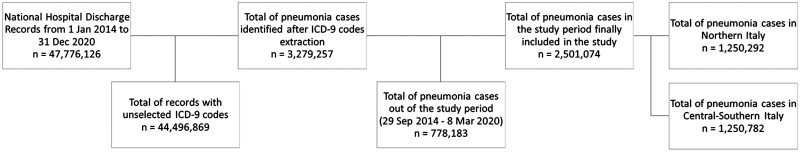
Record selection of the hospitalizations included in this study, Italy, 29 September 2014–8 March 2020.

From 2014 to 2020, a total of 47 776 126 hospitalizations (excluding day hospitals) were reported. By filtering the data according to the ICD-9-CM codes of interest (see Supplementary Table S1), a total of 3 279 257 hospitalizations with pneumonia were identified, of which, 2 501 074 (1 250 292 in Northern Italy and 1 250 782 in Central-Southern Italy) were in the study period (29 September 2014–8 March 2020) (Fig. 1).

**Figure 2.**
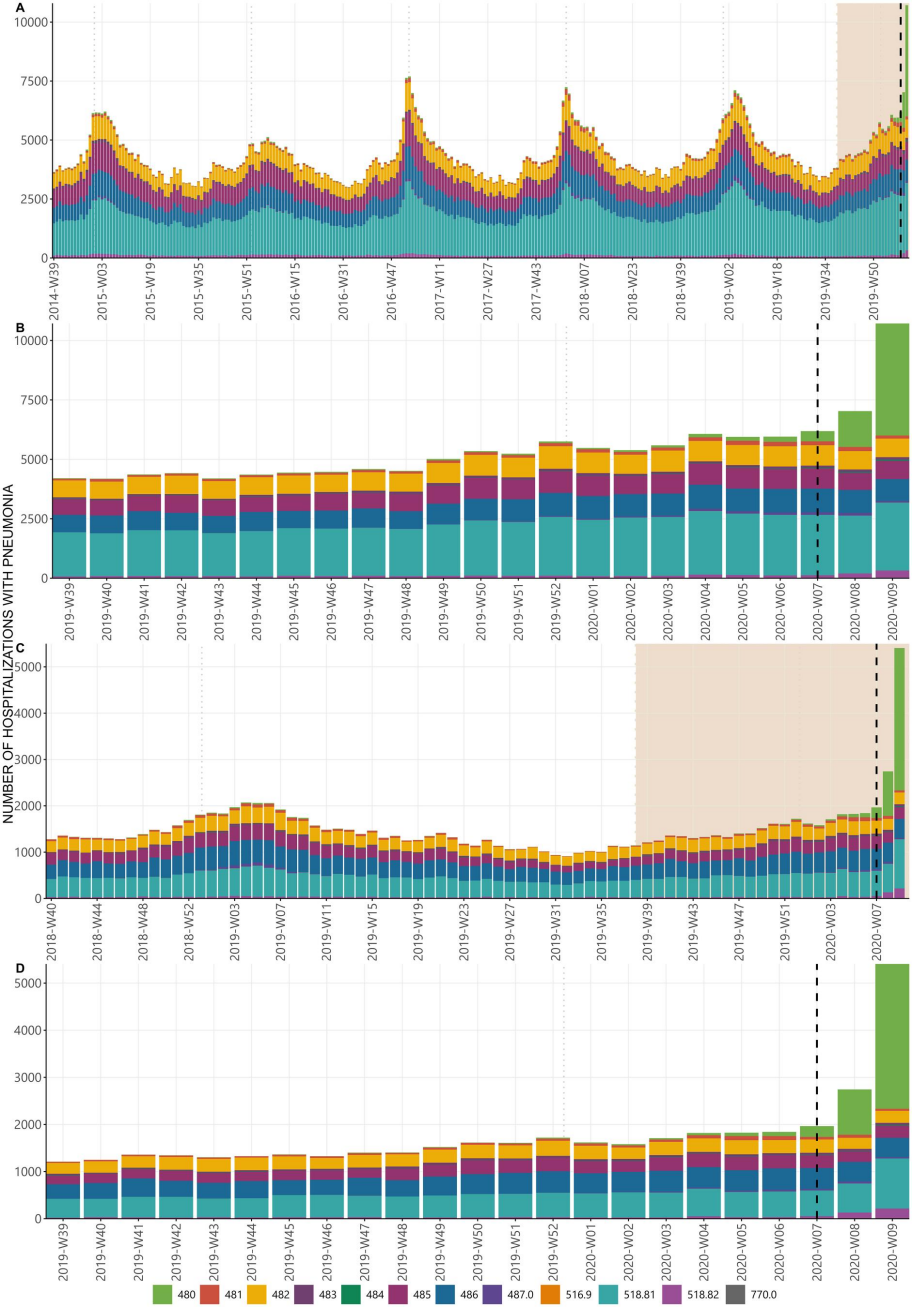
Weekly observed hospitalizations with a pneumonia ICD-9-CM code (any position): (A) Observed value from 29 September 2014–8 March 2020 in ‘Northern Italy’, (B) Same as (A) focusing on the period from 30 September 2019–08 March 2020, (C) observed value from 1 October 2018–8 March 2020 in ‘Lombardy Region’, (D) Same as (C) focusing on the period from 30 September 2019–08 March 2020. Hospital discharge record system, Italy, 29 September 2014–8 March 2020. 480—viral pneumonia; 481—pneumococcal pneumonia; 482—other bacterial pneumonia; 483—pneumonia due to other specified organisms; 484—pneumonia in infectious diseases classified elsewhere; 485—bronchopneumonia with unspecified organisms; 486—pneumonia with unspecified organisms; 487.0—influenza with pneumonia; 516.9—unspecified alveolar and parietoalveolar pneumonopathy; 518.81—acute respiratory failure; 518.82—other pulmonary insufficiency not elsewhere classified; 770.0—congenital pneumonia. In order to facilitate graphical representation, the data has been formatted according to the isoweek convention. The analysis has correctly accounted for any weeks that fall between the end of 1 year and the beginning of the next. The rectangle in panel A indicates the period shown in more detail in panel B (30 September 2019–08 March 2020). The vertical dashed line indicates the first autochthonous COVID-19 case diagnosis in Italy. The vertical dotted light lines indicate the 1 January of each year.

In Fig. 2A, we show the weekly observed hospitalizations with pneumonia during the study period for Northern Italy. We observe that the trend shows a similar periodicity and clear seasonal pattern in each year considered in the analysis, with numbers increasing at the beginning of the ILI season (October–November), peaking in January and then progressively declining. Higher fall/winter peaks can be observed in 2016/17 season; further, a small peak can be observed with the opening of each school year in September. In the 2019/20 season, the main peak was on average initially lower than in previous years. Overall, hospitalizations with pneumonia increased rapidly after the first diagnosed autochthonous case of COVID-19 (20 February 2020). The distribution of the number of hospitalizations with pneumonia for each ICD-9-CM code appears stable over time, except for a slight increase in ICD-9-CM code 480 (viral pneumonia) in the two weeks preceding the first detected local case and a substantial increase afterwards (Fig. 2B). Fig. 2C and D focuses on Lombardy over the period 2018–2020, where the increase in hospitalizations for viral pneumonia was similar to that reported in Northern Italy.

Analysing the trend in the provinces of Lombardy for the period 2018–2020, we see that for all pneumonia hospitalizations, before the date of the first autochthonous case, there was a trend of the cases in line with that observed in the previous years (Supplementary Fig. S2). When evaluating the hospitalizations associated only to viral pneumonia. a rise in the number of hospitalizations for viral pneumonia can be observed starting 2 weeks before the first autochthonous case was diagnosed in the province of Bergamo and Lodi (Supplementary Fig. S3). In contrast, there was not an increase before the first autochthonous case was diagnosed in other provinces (data not shown).

A similar seasonal trend is observed in all Regions/APs from 2018 to 2020 (Supplementary Fig. S4). The increase of pneumonia hospitalizations and particularly those associated to viral pneumonia before the date of the first autochthonous case does not appear to occur in Central-Southern Italy. Even after the first autochthonous case, the overall rise in pneumonia hospitalizations is lower in Southern-Central than in Northern Italy (Supplementary Fig. S5). The trend for the entire Italian territory is shown in Supplementary Fig. S6.

**Figure 3.**
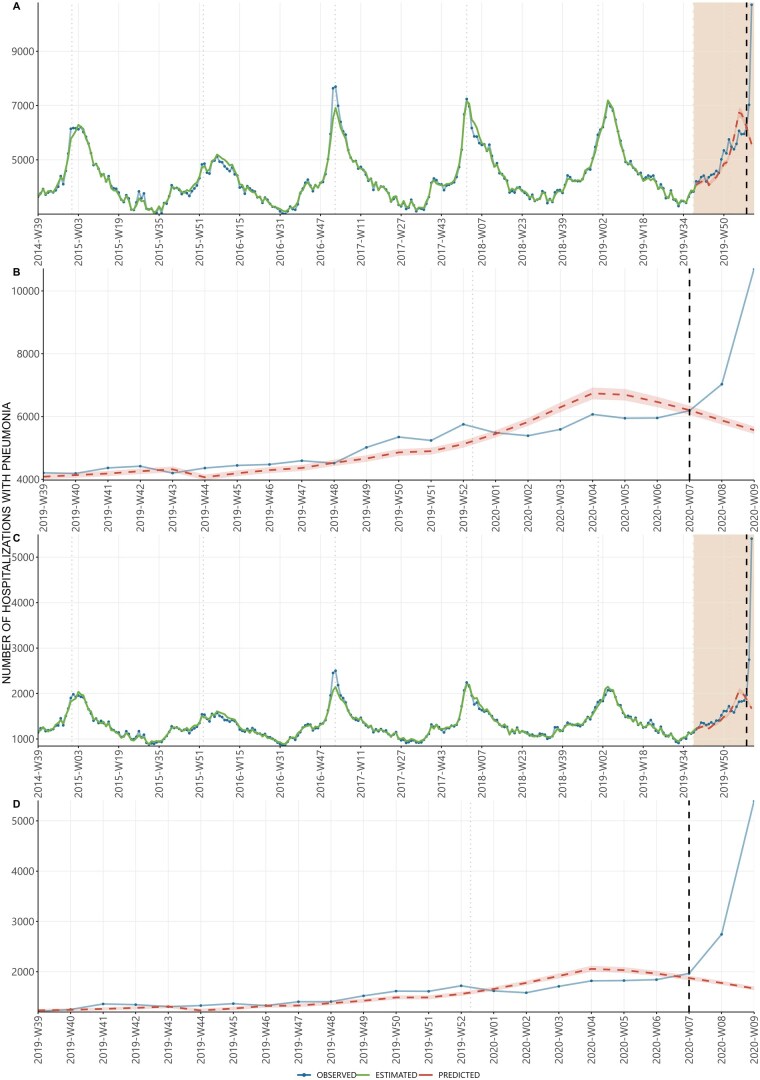
Weekly number pneumonia hospitalizations: (A) Observed, estimated and predicted value during the study period (from 29 September 2014–29 September 2019) in Northern Italy, (B) observed and predicted value from the beginning of 2019 autumn season to the end of the study (from 30 September 2019–8 March 2020) in Northern Italy, (C) observed, estimated and predicted value during the study period (from 29 September 2014 to 29 September 2019) in Lombardy Region, (D) observed and predicted value from the beginning of 2019 autumn season to the end of the study (from 30 September 2019 to 8 March 2020) in Lombardy Region. Hospital discharge record system, Italy, 29 September 2014–8 March 2020. In order to facilitate graphical representation, the data has been formatted according to the isoweek convention. The analysis has correctly accounted for any weeks that fall between the end of one year and the beginning of the next. The rectangle in panel A indicates the period shown in more detail in panel B (30 September 2019–08 March 2020). The vertical dashed line indicates the first autochthonous COVID-19 case diagnosis in Italy. The vertical dotted light lines indicate the 1 January of each year.

The negative binomial model applied to Northern Italy data shows that the weekly expected pneumonia hospitalizations fits overall well with the observed hospitalizations pattern (Fig. 3A) until week 07-2020. Afterwards, as highlighted in Fig. 3B, the observed cases after week 07-2020 were largely higher than those expected. In particular, starting from two weeks before the date of first autochthonous case, the model predicted a descending number of hospitalizations while observations showed a trend inversion with an increase that became progressively more pronounced a week before the first local case detection. Consistent results are observed on the model results obtained for the Lombardy Region (Fig. 3C and D), and more locally within Lombardy for the Bergamo and Lodi provinces (Supplementary Figs S7 and S8).

The analysis limited to viral pneumonia cases (ICD-9-CM code 480) in Lombardy and Bergamo led to similar conclusions (Supplementary Figs S9 and S10). Regarding the province of Lodi, weekly expected cases of viral pneumonia hospitalizations were just below one since the beginning of 2020; in the last 2 weeks of January 2020, there were 3 cases; 2 weeks and 1 week before 20 February 2020 there were 11 and 24 cases, corresponding to more than 10 and 25 times of the expected cases (Supplementary Fig. S11).

We calculated the weekly difference between observed and predicted admission, but for better readability of the results, these are reported in aggregate form (Table 1). January 2020 (from 2020-W01 to 2020-W04) is the month with the highest overestimation (1777 fewer observed admissions than predicted, −7.9%). After the first autochthonous case (2020-W08 to 2020-W09), we observed around one and a half times the expected admissions (35.5% more).

**Table 1.** Monthly number of observed, predicted, and percentage of hospitalizations with pneumonia diagnosis up to 29 September 2019 (Northern Italy, from 2019-W39 to 2020-W09)

Period	Observed (*A*)	Predicted (*B*)	**95% CI** ^a^	Difference (*A*−*B*)	**Percentage** ^b^ (*A*−*B*)*100/(*A*)
2019-W39 2019-W43	21 379	20 986	20 549–21 403	393	1.8%
2019-W44 2019-W47	17 871	16 918	16 542–17 288	953	5.3%
2019-W48 2019-W52	25 871	24 070	23 530–24 599	1801	7.0%
2020-W01 2020-W04	22 533	24 310	23 722–24 886	−1777	−7.9%
2020-W05 2019-W07	18 088	19 361	18 871–19 850	−1273	−7.0%
2020-W08 2020-W09	17 745	11 444	11 200–11 688	6301	35.5%

a Confidence interval (CI) obtained using bootstrap method.

b Percentage difference of hospitalizations.

Lastly, the retrospective analysis on negative ILI test in Lombardy Region, revealed that in the 2019/2020 season the increase began in mid-February, remaining consistent with previous seasons until mid-March; notably, the preceding season, 2018/2019, exhibited an even lower seasonal minimum in the percentage of negative ILI tests (Supplementary Fig. S12).

## Discussion

The main challenge in the rapid detection of COVID-19 at the start of pandemic circulation was the similarity of clinical presentation with other influenza-like illnesses (ILI) [30]. This included mild as well as severe cases being pneumonia a common complication in both COVID-19 and influenza infections [31]. This combined with the limited availability of diagnostics that were mostly based on in house molecular assays that required high level expertise and processing time. Therefore, sensitivity and specificity of surveillance were hard to optimize from the start. The enhanced Italian SARI surveillance system, implemented before the declaration of the COVID-19 pandemic, identified over 200 cases initially suspected for SARS CoV-2 between the last decade of January and 20 February 2020, of which only three were confirmed, all travel related. Despite having no history of international travel or contact with people who tested positive for the virus, an unusually severe case of atypical pneumonia hospitalized in Northern Italy was tested and found positive for SARS-CoV-2 on 20th of February marking the first locally acquired case diagnosed [32].

In order to explore whether previous signals of increased hospitalizations for pneumonia were present between October 2019 and early February 2020 we used a classical signal detection approach in epidemiology by comparing current trends with trends observed to the same period of previous years.

Between September 2014 and February 2020 trends in hospitalizations with pneumonia, showed a clear seasonal pattern over the years and a relatively stable distribution in terms of the aetiology. Our models effectively captured the weekly pattern of pneumonia hospitalizations and successfully predicted the increase in cases at the beginning of the 2019/20 influenza season. However, it also overestimated the number of hospitalizations in January and the first half of February 2020 [33]. This finding is coherent with an unusually mild influenza season as observed by the ILI surveillance at that time, leading to lower overall case-loads of pneumonia hospitalizations.

Our results show that, overall, hospitalizations for pneumonia in the weeks 39-2019 to week 07-2020 did not increase above the threshold of previously observed seasonal hospitalizations until 2 weeks after the detection of the first locally acquired COVID-19 case in Italy. This finding is consistent with the observed excess of influenza cases and deaths in the season [34]. When analysing the pneumonia hospitalizations by type of ICD-9-CM code, while the expected threshold of pneumonia hospitalizations was not breached, it was possible to observe a subthreshold relative increase of hospitalizations related to viral pneumonia only in the two weeks immediately preceding the first detection of locally-acquired SARS-CoV-2. This finding does not support the hypothesis of a large-scale undetected circulation of the virus with numerous severe cases of infection occurring undetected from the last months of 2019 to early February 2020.

As the thresholds were not breached, signal detection would not have therefore been triggered even if HDRs had been available in real time and not consolidated in the following years.

The subthreshold relative increase, if detected locally, could have been reasonably interpreted as fluctuations that did not determine overall an excess case-load of expectable pneumonia hospitalizations [7].

Shifting to the Lombardy region, the area most affected by the first wave of COVID-19 in Italy [27], this slight increase in hospitalization with pneumonia was appreciated in the 2 weeks prior to 20 February 2020, as retrospectively observed during the first month of the epidemic [7]. At provincial level, only the data for the provinces of Bergamo and Lodi show an increase of pneumonia hospitalizations in the preceding period, but also in this case a more substantial increase was only observed in the weeks following the first local case detection [33]. The findings remained consistent even when considering only hospitalizations with viral pneumonia (ICD-9-CM code 480) in these areas.

As assessed by the Italian Epidemic Intelligence Network at the time [35], the mild presentation of COVID-19 among young population, combined with lower-than-expected hospitalization rates for respiratory infections, might have masked its initial spread and impact in Lombardy.

However, even if the incidence of severe COVID-19 in Northern Italy was not high enough to impact substantially on hospitalization trends at the time of the first case detection, our findings suggest that hospital discharge data, if available in a more-timely manner might support early detection of changes in hospitalization trends by looking and code specific conditions (such as viral pneumonia) at subnational level.

This study has several limitations. By using discharge data, we cannot exclude the possibility that viral circulation may have been present in individuals less likely to develop severe disease requiring hospitalization. This could be partly overcome by evaluating other health records. Murtas et al. used emergency department access data to assess the onset of the COVID-19 peak [36]. Despite using different data, this study used the same proxy as ours (severity of illness and hospitalization) and, consistent with our findings, the authors found no increase in pneumonia-related emergency department access in the area of Milan (Lombardy). It must also be taken into account that, due to the initial lack of COVID-19 codes in the ICD-9-CM system, multiple codes were used for early COVID-19 patient admissions, making it difficult to identify them before the coding guidelines were established. Finally, although our data show an increase in hospitalization with viral pneumonia 2 weeks before the index case, it should be noted that we use hospital admission dates as the temporal proxy for onset. However, we cannot exclude the possibility that some patients admitted on a certain date might have tested positive for COVID-19 during their hospital stay and developed pneumonia over time. This could have led to some cases being assigned a date that preceded the true onset of disease.

In conclusion our findings show that there were no signals of an unusual increase in hospitalizations with pneumonia before the first identification of local SARS-CoV-2 transmission in Northern Italy. Although slight relative increases in viral pneumonia hospitalizations occurred in the weeks immediately preceding the first locally transmitted case, overall hospitalizations did not exceed those observed in previous years. Thanks to retrospective recoding we now know that this slight relative increase in viral pneumonia hospitalizations was due to SARS-CoV-2, but this could have been initially attributed to a tail of influenza infections as the season had been particularly mild in the previous months. This interpretation, given the knowledge available at the time on the epidemiology of SARS-CoV-2, would have been the most plausible.

## Supplementary Material

ckaf137_Supplementary_Data

## Data Availability

The data underlying this article were provided by the Italian Ministry of Health by permission. The data analysed in the current study are not publicly available due to privacy policies.
