# Supplementary material for: Was severe SARS-CoV-2 substantially spreading in Northern Italy before its first detection in February 2020? An evaluation of pneumonia-associated hospitalization trends from September 2014 to February 2020
Source: Eur J Public Health. 2025 Aug 4;35(5):1050–7. doi: 10.1093/eurpub/ckaf137 (PMC12529277; doi:10.1093/eurpub/ckaf137)
Supplement: ckaf137_Supplementary_Data [file ckaf137_supplementary_data.zip › ckaf137_Supplementary_Data/ejph-2024-11-om-0818-File006.docx]

**Supplementary Table S1** – Selected ICD-9-CM codes (categories, subcategories, subclassifications) and their descriptions

| **Selected ICD-9-CM codes (categories, subcategories, subclassifications) and their descriptions** | | | |
| --- | --- | --- | --- |
| **480** | **Viral pneumonia** | | |
|  | 480.0 | Pneumonia due to adenovirus | |
|  | 480.1 | Pneumonia due to respiratory syncytial virus | |
|  | 480.2 | Pneumonia due to parainfluenza virus | |
|  | 480.3 | Pneumonia due to SARS-associated coronavirus | |
|  | 480.4* | Pneumonia in COVID-19 | |
|  |  | 480.41* | Pneumonia in COVID-19, virus identified |
|  |  | 480.42* | Pneumonia in COVID-19, virus unidentified |
|  | 480.8 | Pneumonia due to other virus not elsewhere classified | |
|  | 480.9 | Viral pneumonia, unspecified | |
| **481** | **Pneumococcal pneumonia [Streptococcus pneumoniae pneumonia]** | | |
| **482** | **Other bacterial pneumonia** | | |
|  | 482.0 | Pneumonia due to Klebsiella pneumoniae | |
|  | 482.1 | Pneumonia due to Pseudomonas | |
|  | 482.2 | Pneumonia due to Hemophilus influenzae [H. influenzae] | |
|  |  | 482.30 | Pneumonia due to Streptococcus, unspecified |
|  |  | 482.31 | Pneumonia due to Streptococcus, group A |
|  |  | 482.32 | Pneumonia due to Streptococcus, group B |
|  |  | 482.39 | Pneumonia due to other Streptococcus |
|  |  | 482.40 | Pneumonia due to Staphylococcus, unspecified |
|  |  | 482.41 | Methicillin susceptible pneumonia due to Staphylococcus aureus |
|  |  | 482.42 | Methicillin resistant pneumonia due to Staphylococcus aureus |
|  |  | 482.49 | Other Staphylococcus pneumonia |
|  |  | 482.81 | Pneumonia due to anaerobes |
|  |  | 482.82 | Pneumonia due to escherichia coli [E. coli] |
|  |  | 482.83 | Pneumonia due to other gram-negative bacteria |
|  |  | 482.84 | Pneumonia due to Legionnaires' disease |
|  |  | 482.89 | Pneumonia due to other specified bacteria |
|  | 482.9 | Bacterial pneumonia, unspecified | |
| **483** | **Pneumonia due to other specified organism** | | |
|  | 483.0 | Pneumonia due to mycoplasma pneumoniae | |
|  | 483.1 | Pneumonia due to chlamydia | |
|  | 483.8 | Pneumonia due to other specified organism | |
| **484** | **Pneumonia in infectious diseases classified elsewhere** | | |
|  | 484.1 | Pneumonia in cytomegalic inclusion disease | |
|  | 484.3 | Pneumonia in whooping cough | |
|  | 484.5 | Pneumonia in anthrax | |
|  | 484.6 | Pneumonia in aspergillosis | |
|  | 484.7 | Pneumonia in other systemic mycoses | |
|  | 484.8 | Pneumonia in other infectious diseases classified elsewhere | |
| **485** | **Bronchopneumonia, organism unspecified** | | |
| **486** | **Pneumonia, organism unspecified** | | |
|  | 487.0 ^#^ | Influenza with pneumonia | |
|  | **516.9** ^#^ | **Unspecified alveolar and parietoalveolar pneumonopathy** | |
|  |  | **518.81** ^o^ | **Acute respiratory failure** |
|  |  | **518.82** ^o^ | **Other pulmonary insufficiency, not elsewhere classified** |
|  | **770.0** ^#^ | **Congenital pneumonia** | |

* New ICD-9-CM code;

^#^ selected only the subcategories listed;

^o^ selected only the subclassifications listed.
