# Supplementary material for: Was severe SARS-CoV-2 substantially spreading in Northern Italy before its first detection in February 2020? An evaluation of pneumonia-associated hospitalization trends from September 2014 to February 2020
Source: Eur J Public Health. 2025 Aug 4;35(5):1050–7. doi: 10.1093/eurpub/ckaf137 (PMC12529277; doi:10.1093/eurpub/ckaf137)
Supplement: ckaf137_Supplementary_Data [file ckaf137_supplementary_data.zip › ckaf137_Supplementary_Data/ejph-2024-11-om-0818-File007.docx]

**Supplementary Figure S1 –** Weekly number of diagnosis and hospitalizations of SARS-CoV-2 reported to the Italian COVID-19 Integrated Surveillance System. Italy, 27 January 2020 – 8 March 2020


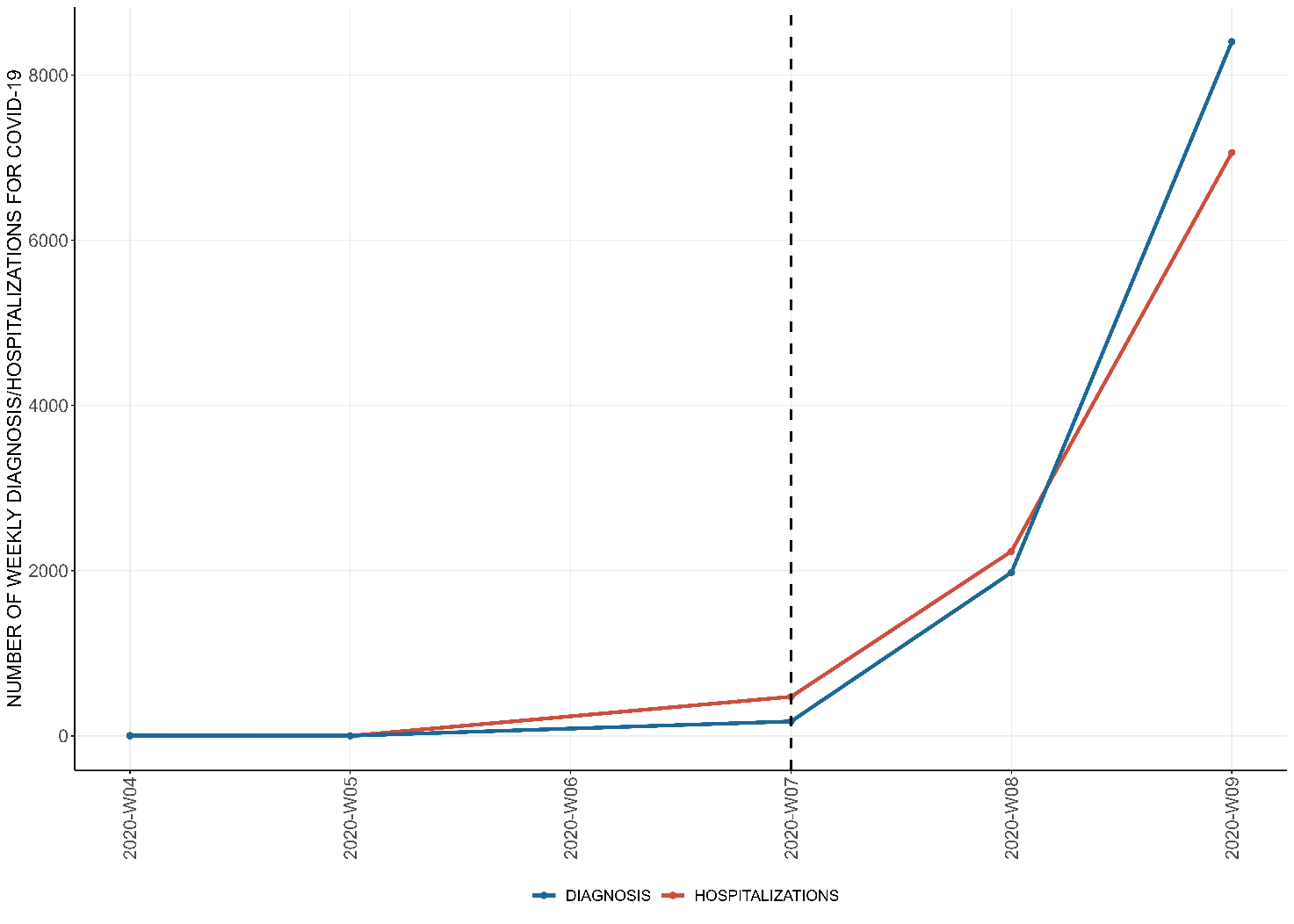


In order to facilitate graphical representation, the data has been formatted according to the isoweek convention. The analysis has correctly accounted for any weeks that fall between the end of one year and the beginning of the next. The vertical dashed line indicates the first autochthonous COVID-19 case diagnosis in Italy.

**Supplementary Figure S2** – Weekly observed hospitalizations with pneumonia in Lombardy provinces, 30 September 2019 – 8 March 2020. Hospital discharge record system, Italy, 30 September 2019 – 8 March 2020


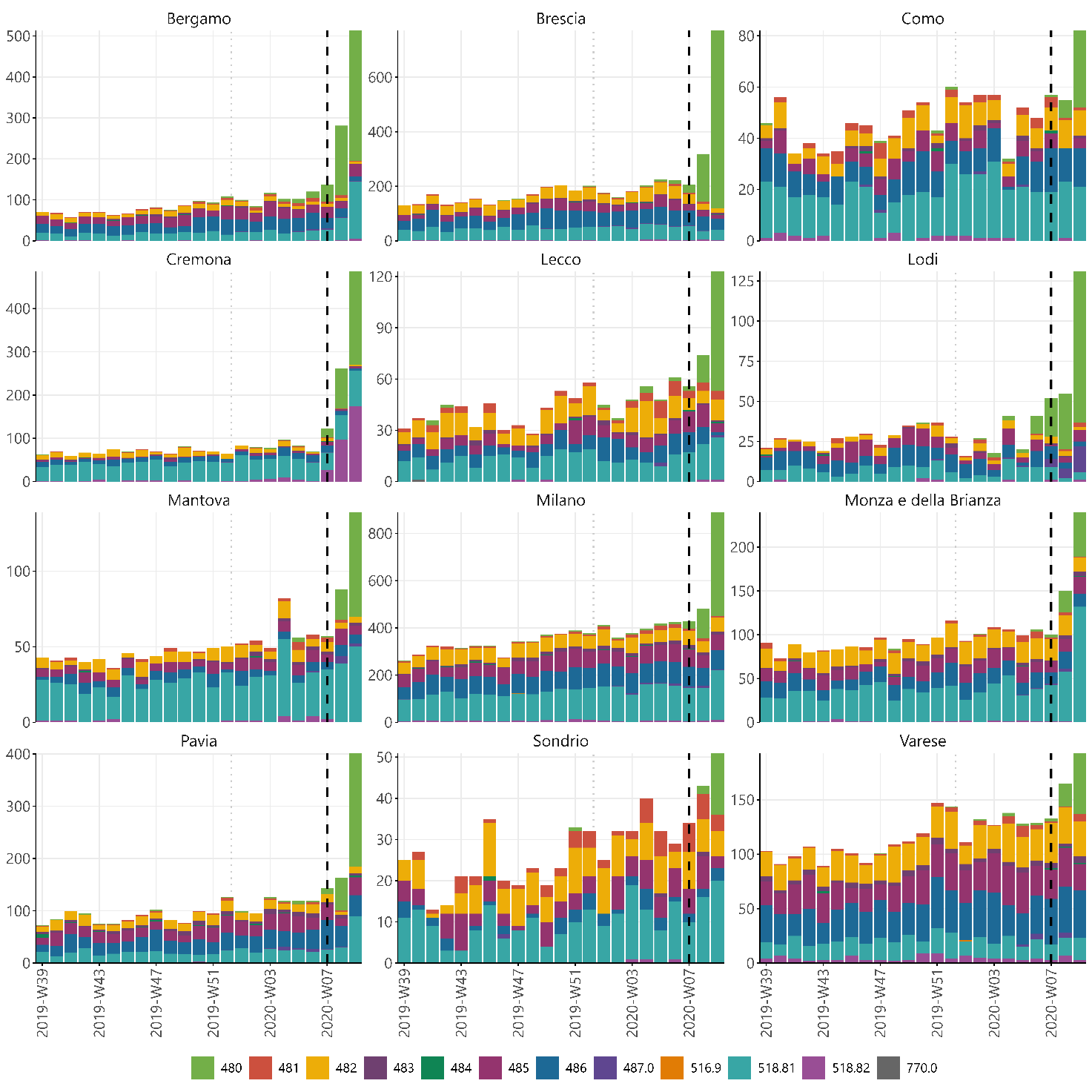


480 - viral pneumonia; 481 - pneumococcal pneumonia; 482 - other bacterial pneumonia; 483 - pneumonia due to other specified organisms; 484 - pneumonia in infectious diseases classified elsewhere; 485 - bronchopneumonia with unspecified organisms; 486 - pneumonia with unspecified organisms; 487.0 - influenza with pneumonia; 516.9 - unspecified alveolar and parietoalveolar pneumonopathy; 518.81 - acute respiratory failure; 518.82 - other pulmonary insufficiency not elsewhere classified; 770.0 - congenital pneumonia

In order to facilitate graphical representation, the data has been formatted according to the isoweek convention. The analysis has correctly accounted for any weeks that fall between the end of one year and the beginning of the next. The vertical dashed line indicates the first autochthonous COVID-19 case diagnosis in Italy. The vertical dotted light lines indicate the 1 January of each year.

**Supplementary Figure S3** – Weekly observed hospitalizations with a pneumonia ICD-9-CM code (any position): A) Observed value from 29 September 2014 to 08 March 2020 in Bergamo province, B) Same as A) focusing on the period from 30 September 2019 to 08 March 2020, C) Observed value from 29 September 2014 to 8 March 2020 in Lodi province, D) Same as C) focusing on the period from 30 September 2019 to 08 March 2020. Hospital discharge record system, Italy, 29 September 2014 – 8 March 2020


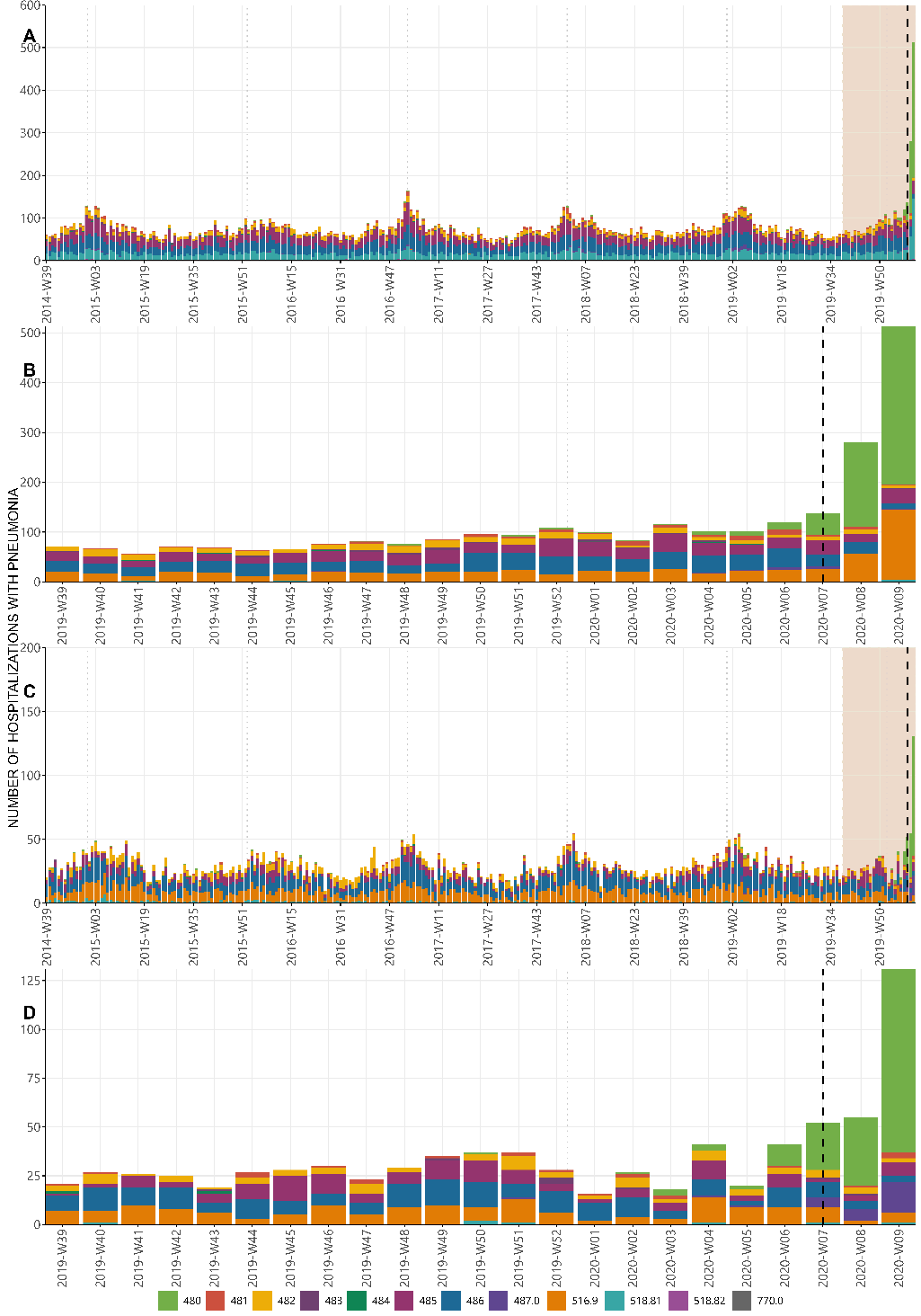


480 - viral pneumonia; 481 - pneumococcal pneumonia; 482 - other bacterial pneumonia; 483 - pneumonia due to other specified organisms; 484 - pneumonia in infectious diseases classified elsewhere; 485 - bronchopneumonia with unspecified organisms; 486 - pneumonia with unspecified organisms; 487.0 - influenza with pneumonia; 516.9 - unspecified alveolar and parietoalveolar pneumonopathy; 518.81 - acute respiratory failure; 518.82 - other pulmonary insufficiency not elsewhere classified; 770.0 - congenital pneumonia

In order to facilitate graphical representation, the data has been formatted according to the isoweek convention. The analysis has correctly accounted for any weeks that fall between the end of one year and the beginning of the next.

The rectangle in panel A indicates the period shown in more detail in panel B (30 September 2019 - 08 March 2020). The vertical dashed line indicates the first autochthonous COVID-19 case diagnosis in Italy. The vertical dotted light lines indicate the 1 January of each year.

**Supplementary Figure S4** – Weekly observed hospitalizations with pneumonia in **Italian Regions/APs**, 1 October 2018 - 8 March 2020. Hospital discharge record system, Italy, 1 October 2018 – 8 March 2020


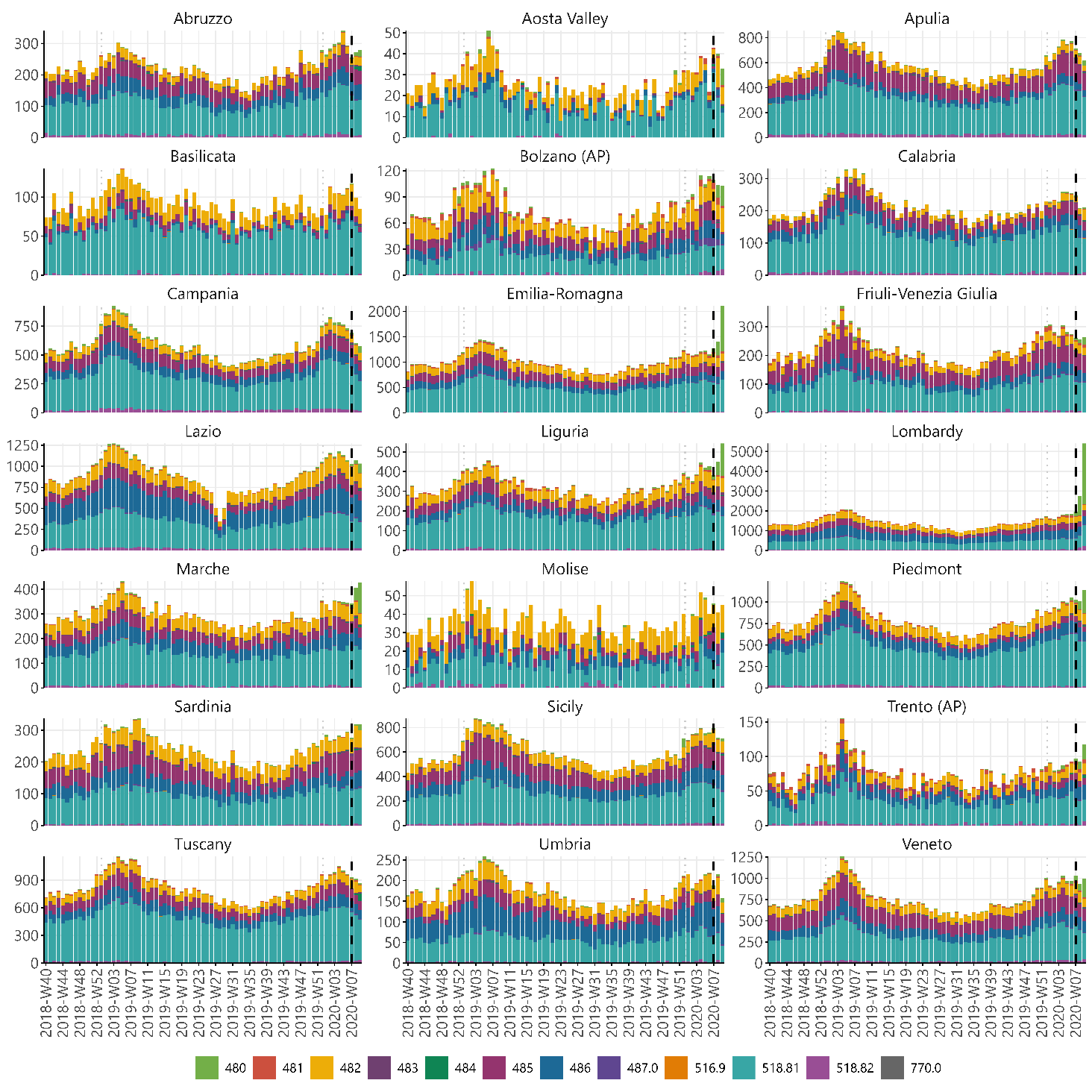


480 - viral pneumonia; 481 - pneumococcal pneumonia; 482 - other bacterial pneumonia; 483 - pneumonia due to other specified organisms; 484 - pneumonia in infectious diseases classified elsewhere; 485 - bronchopneumonia with unspecified organisms; 486 - pneumonia with unspecified organisms; 487.0 - influenza with pneumonia; 516.9 - unspecified alveolar and parietoalveolar pneumonopathy; 518.81 - acute respiratory failure; 518.82 - other pulmonary insufficiency not elsewhere classified; 770.0 - congenital pneumonia

In order to facilitate graphical representation, the data has been formatted according to the isoweek convention. The analysis has correctly accounted for any weeks that fall between the end of one year and the beginning of the next. The vertical dashed line indicates the first autochthonous COVID-19 case diagnosis in Italy. The vertical dotted light grey lines indicate the 1 January of each year.

**Supplementary Figure S5** – Weekly observed hospitalizations with a pneumonia ICD-9-CM code (any position): A) Observed value from 29 September 2014 to 08 March 2020 in Central-Southern Italy, B) Same as A) focusing on the period from 30 September 2019 to 08 March 2020. Hospital discharge record system, Italy, 29 September 2014 – 8 March 2020


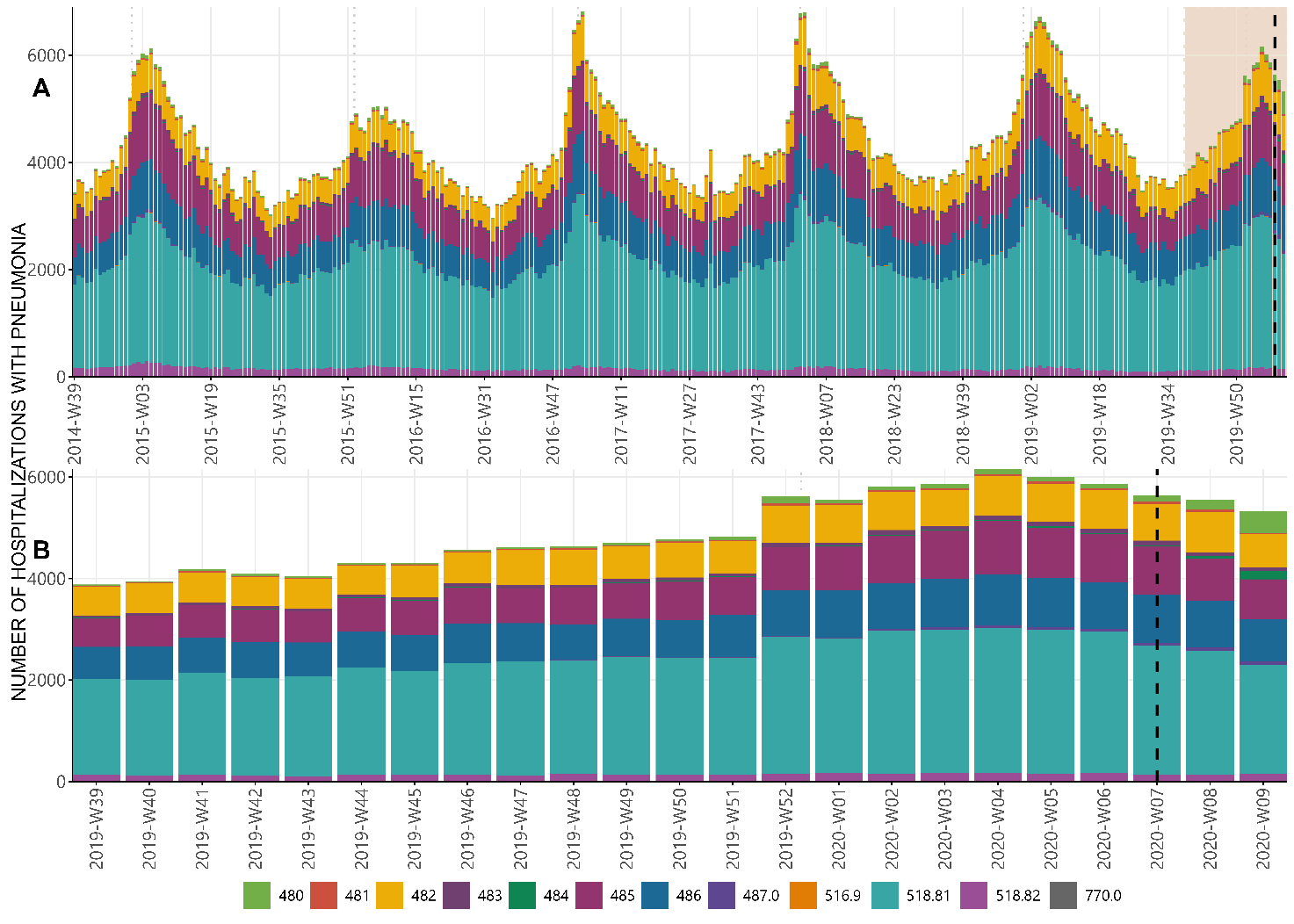


480 - viral pneumonia; 481 - pneumococcal pneumonia; 482 - other bacterial pneumonia; 483 - pneumonia due to other specified organisms; 484 - pneumonia in infectious diseases classified elsewhere; 485 - bronchopneumonia with unspecified organisms; 486 - pneumonia with unspecified organisms; 487.0 - influenza with pneumonia; 516.9 - unspecified alveolar and parietoalveolar pneumonopathy; 518.81 - acute respiratory failure; 518.82 - other pulmonary insufficiency not elsewhere classified; 770.0 - congenital pneumonia

In order to facilitate graphical representation, the data has been formatted according to the isoweek convention. The analysis has correctly accounted for any weeks that fall between the end of one year and the beginning of the next.

The rectangle in panel A indicates the period shown in more detail in panel B (30 September 2019 - 08 March 2020). The vertical dashed line indicates the first autochthonous COVID-19 case diagnosis in Italy. The vertical dotted light lines indicate the 1 January of each year.

**Supplementary Figure S6** – Weekly observed hospitalizations with a pneumonia ICD-9-CM code (any position): A) Observed value from 29 September 2014 to 08 March 2020 in Italy, B) Same as A) focusing on the period from 30 September 2019 to 08 March 2020. Hospital discharge record system, Italy, 29 September 2014 – 8 March 2020


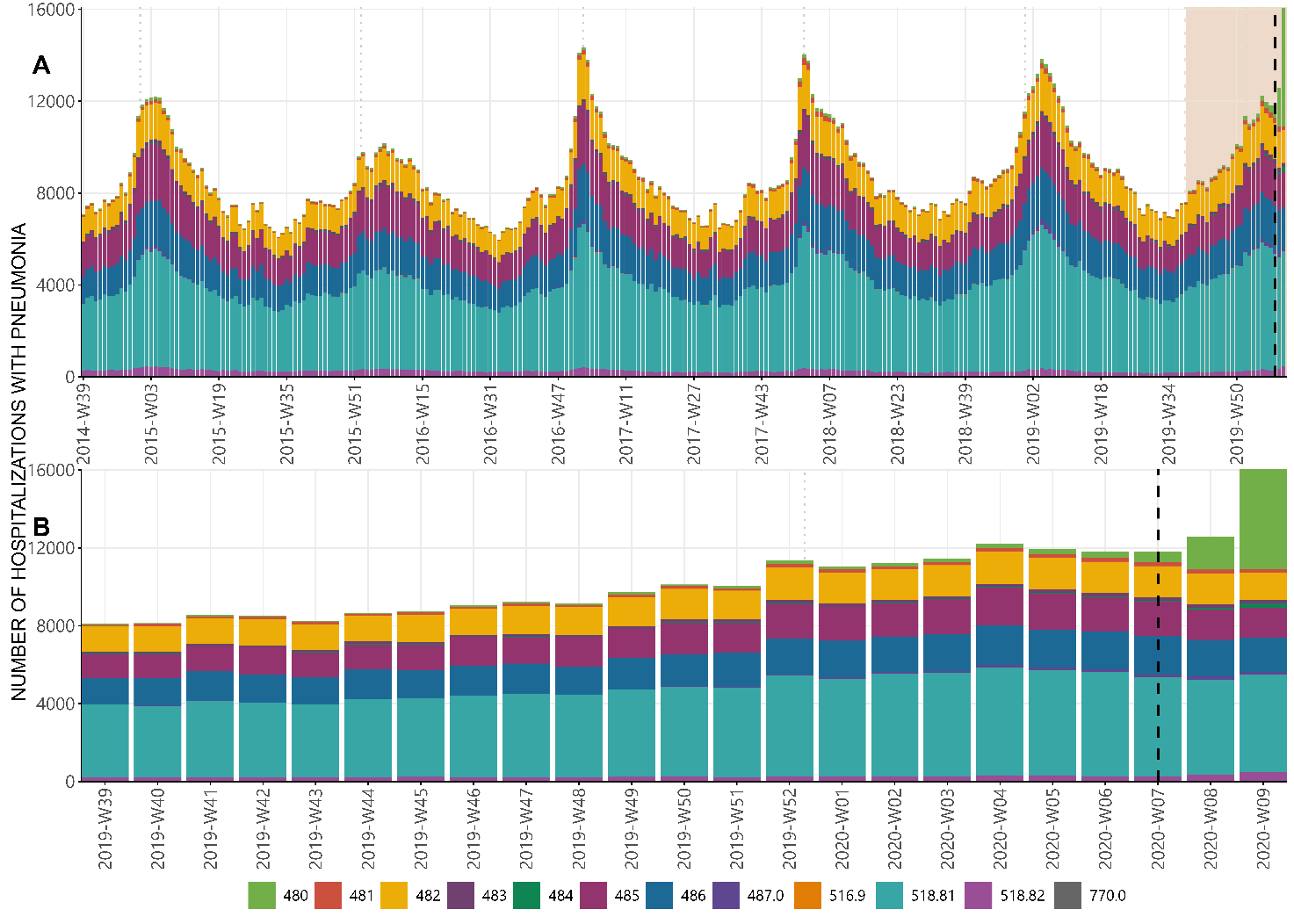


480 - viral pneumonia; 481 - pneumococcal pneumonia; 482 - other bacterial pneumonia; 483 - pneumonia due to other specified organisms; 484 - pneumonia in infectious diseases classified elsewhere; 485 - bronchopneumonia with unspecified organisms; 486 - pneumonia with unspecified organisms; 487.0 - influenza with pneumonia; 516.9 - unspecified alveolar and parietoalveolar pneumonopathy; 518.81 - acute respiratory failure; 518.82 - other pulmonary insufficiency not elsewhere classified; 770.0 - congenital pneumonia

In order to facilitate graphical representation, the data has been formatted according to the isoweek convention. The analysis has correctly accounted for any weeks that fall between the end of one year and the beginning of the next.

The rectangle in panel A indicates the period shown in more detail in panel B (30 September 2019 - 08 March 2020). The vertical dashed line indicates the first autochthonous COVID-19 case diagnosis in Italy. The vertical dotted light lines indicate the 1 January of each year.
