# Supplementary material for: Was severe SARS-CoV-2 substantially spreading in Northern Italy before its first detection in February 2020? An evaluation of pneumonia-associated hospitalization trends from September 2014 to February 2020
Source: Eur J Public Health. 2025 Aug 4;35(5):1050–7. doi: 10.1093/eurpub/ckaf137 (PMC12529277; doi:10.1093/eurpub/ckaf137)
Supplement: ckaf137_Supplementary_Data [file ckaf137_supplementary_data.zip › ckaf137_Supplementary_Data/ejph-2024-11-om-0818-File008.docx]

**Supplementary Figure S7** – Weekly number pneumonia hospitalizations in Italy: A) Observed and estimated value before the 2019 autumn season (from 29 September 2014 to 8 March 2020), B) Observed and predicted value from the beginning of 2019 autumn season to the end of the study (from 30 September 2019 to 8 March 2020). Hospital discharge record system, Italy, 29 September 2014 – 8 March 2020


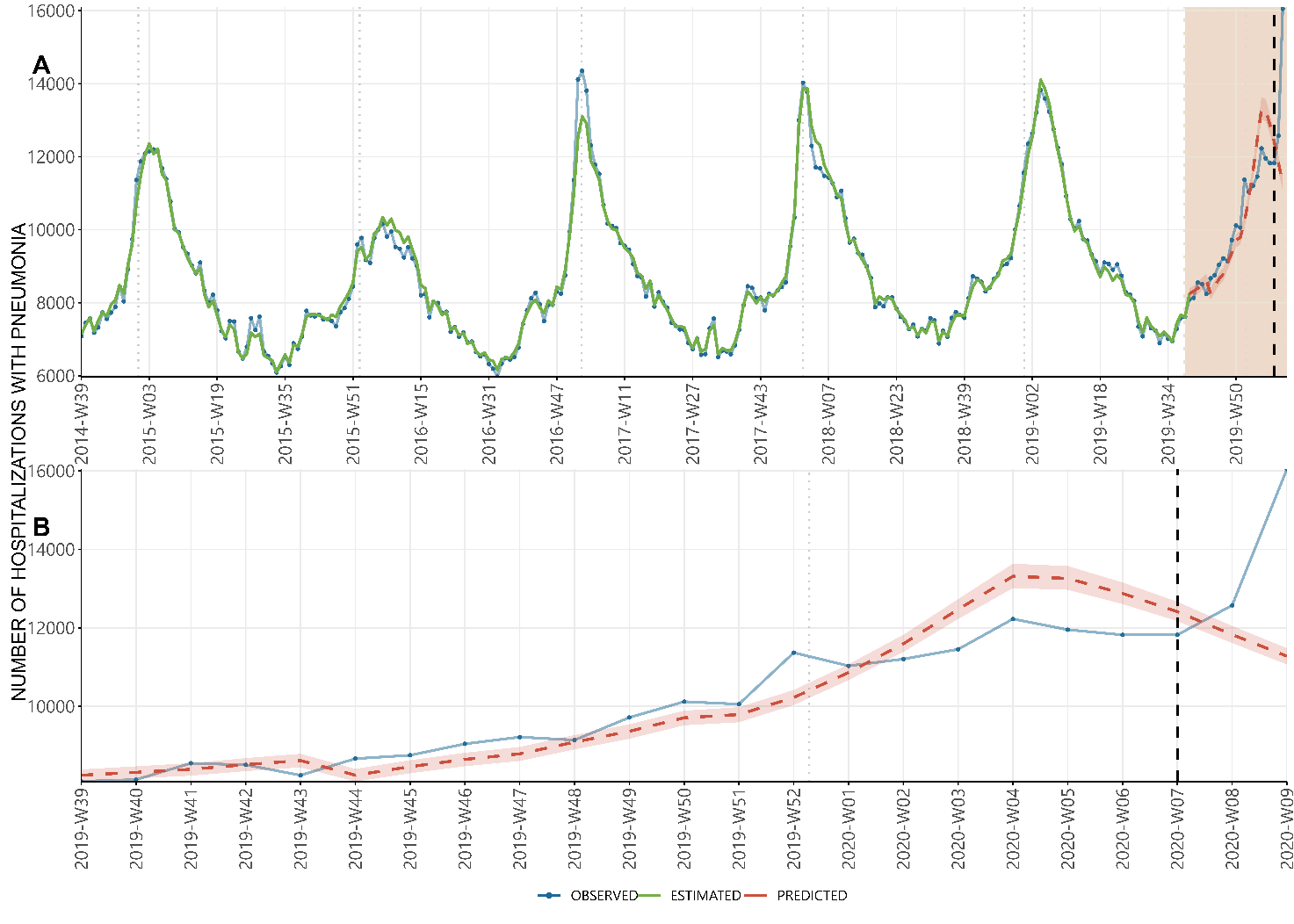


In order to facilitate graphical representation, the data has been formatted according to the isoweek convention. The analysis has correctly accounted for any weeks that fall between the end of one year and the beginning of the next.

The rectangle in panel A indicates the period shown in more detail in panel B (30 September 2019 - 08 March 2020). The vertical dashed line indicates the first autochthonous COVID-19 case diagnosis in Italy. The vertical dotted light lines indicate the 1 January of each year.

**Supplementary Figure S8** – Weekly number pneumonia hospitalizations: A) Observed, estimated and predicted value during the study period (from 29 September 2014 to 29 September 2019) in Bergamo province, B) Observed and predicted value from the beginning of 2019 autumn season to the end of the study (from 30 September 2019 to 8 March 2020) in Bergamo province, C) Observed, estimated and predicted value during the study period (from 29 September 2014 to 29 September 2019) in Lodi province, D) Observed and predicted value from the beginning of 2019 autumn season to the end of the study (from 30 September 2019 to 8 March 2020) in Lodi province. Hospital discharge record system, Italy, 29 September 2014 – 8 March 2020


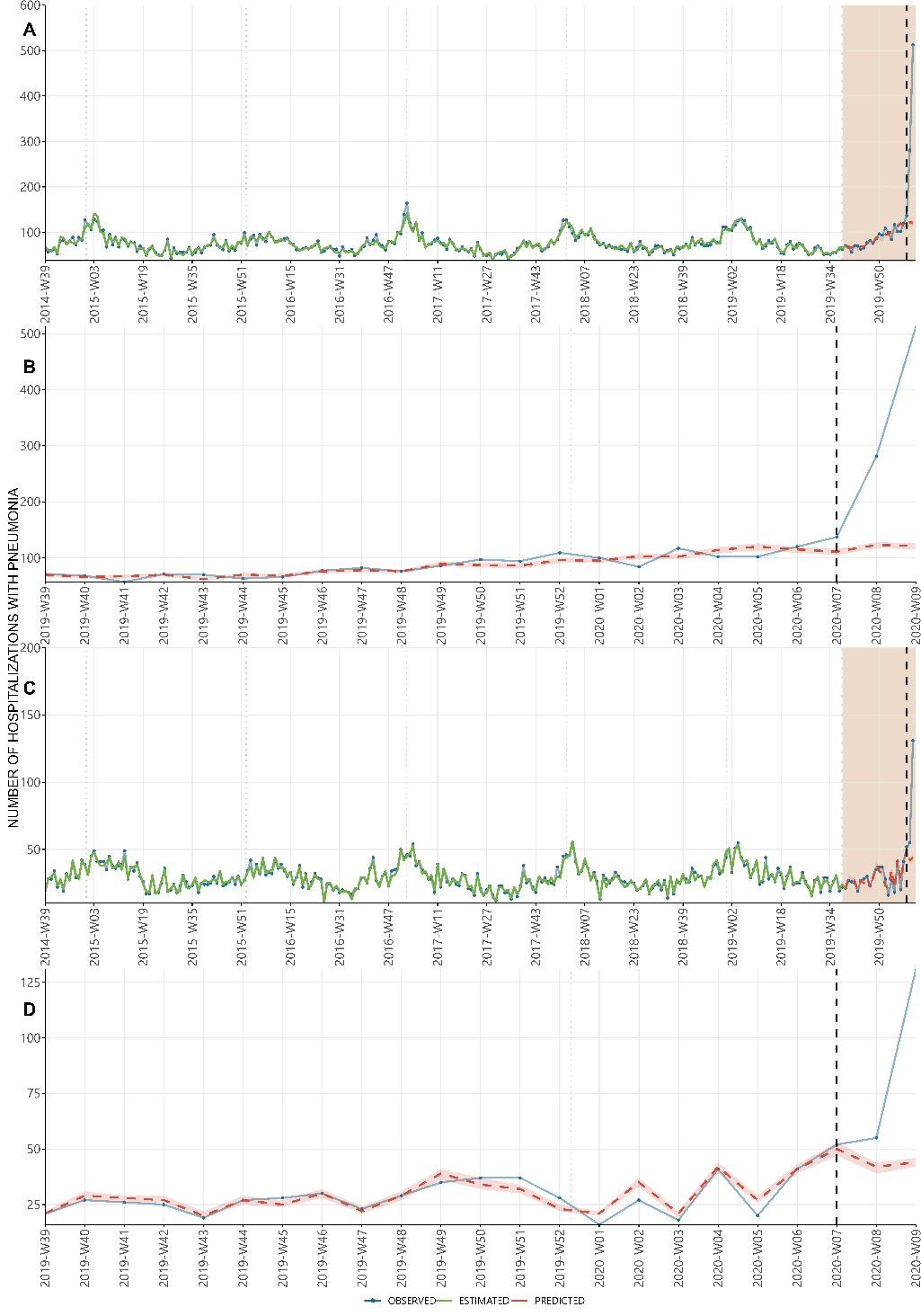


In order to facilitate graphical representation, the data has been formatted according to the isoweek convention. The analysis has correctly accounted for any weeks that fall between the end of one year and the beginning of the next.

The rectangle in panel A indicates the period shown in more detail in panel B (30 September 2019 - 08 March 2020). The vertical dashed line indicates the first autochthonous COVID-19 case diagnosis in Italy. The vertical dotted light lines indicate the 1 January of each year.

**Supplementary Figure S9** – Weekly number of viral pneumonia hospitalizations (ICD-9-CM code 480): A) Observed, estimated and predicted value during the study period (from 29 September 2014 to 29 September 2019) in Lombardy region, B) Observed and predicted value from the beginning of 2019 autumn season to the end of the study (from 30 September 2019 to 8 March 2020) in Lombardy region. Hospital discharge record system, Italy, 29 September 2014 – 8 March 2020


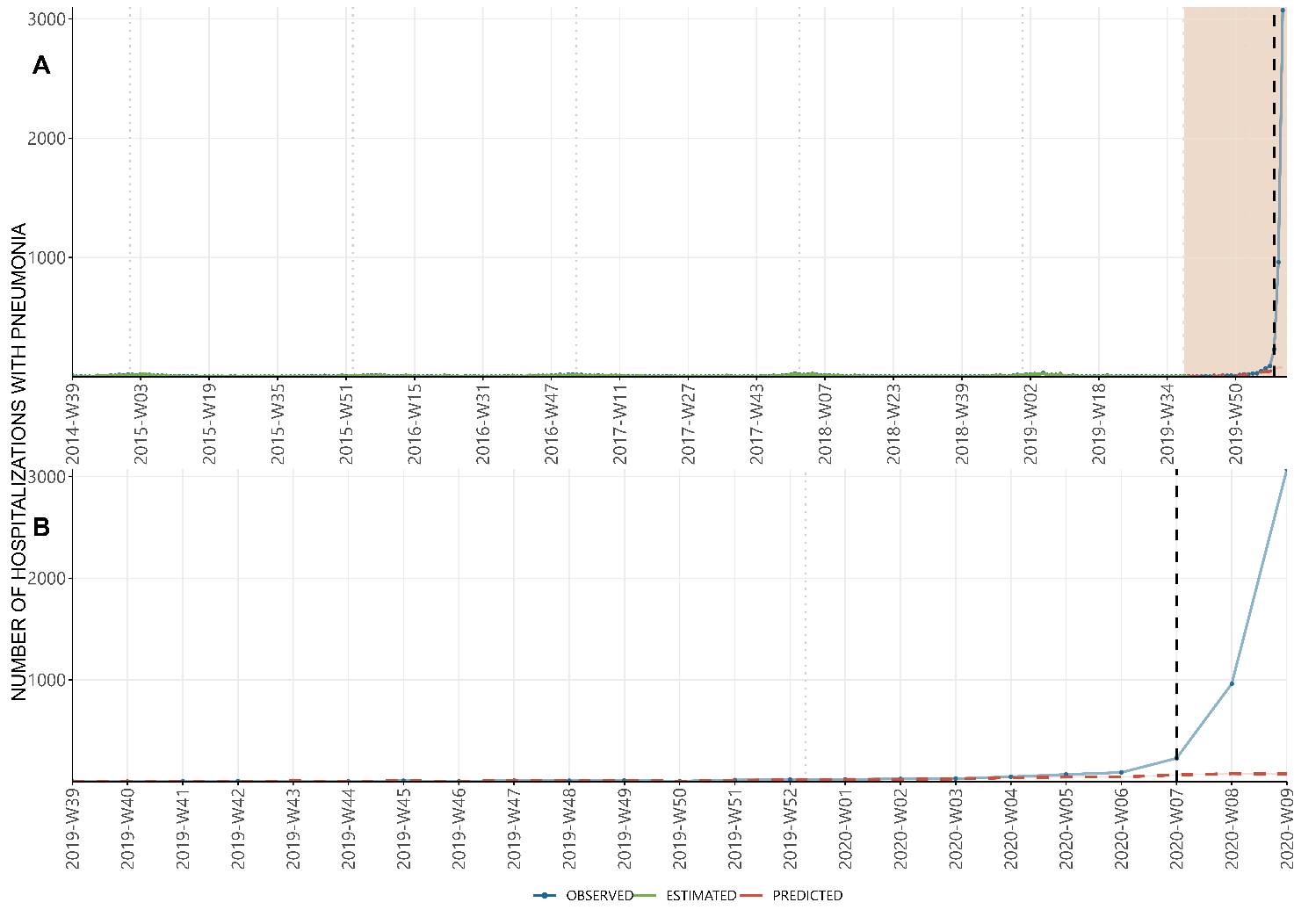


In order to facilitate graphical representation, the data has been formatted according to the isoweek convention. The analysis has correctly accounted for any weeks that fall between the end of one year and the beginning of the next.

The rectangle in panel A indicates the period shown in more detail in panel B (30 September 2019 - 08 March 2020). The vertical dashed line indicates the first autochthonous COVID-19 case diagnosis in Italy. The vertical dotted light lines indicate the 1 January of each year.

**Supplementary Figure S10** – Weekly number viral pneumonia hospitalizations (ICD-9-CM code 480): A) Observed, estimated and predicted value during the study period (from 29 September 2014 to 29 September 2019) in Bergamo province, B) Observed and predicted value from the beginning of 2019 autumn season to the end of the study (from 30 September 2019 to 8 March 2020) in Bergamo province. Hospital discharge record system, Italy, 29 September 2014 – 8 March 2020


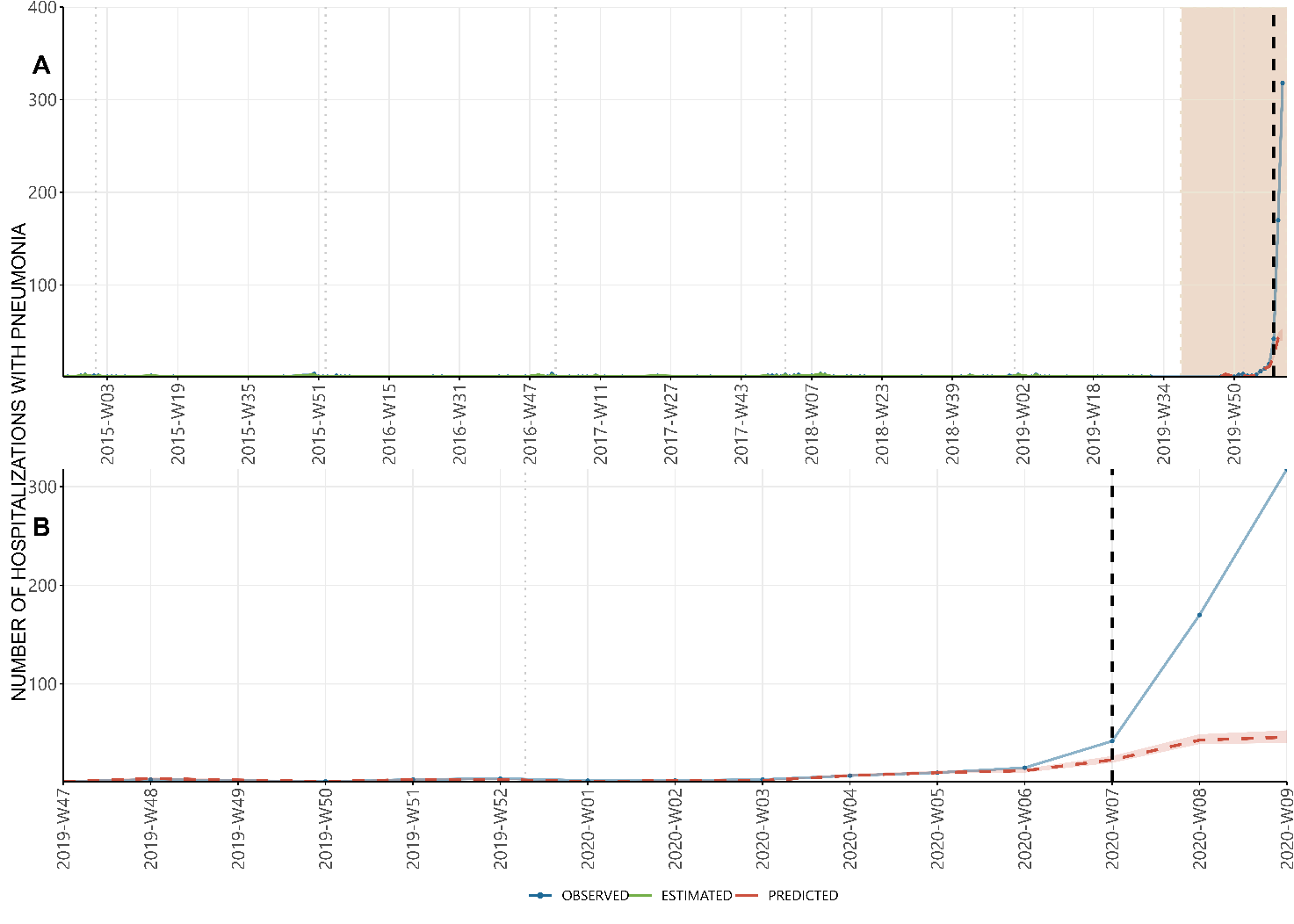


In order to facilitate graphical representation, the data has been formatted according to the isoweek convention. The analysis has correctly accounted for any weeks that fall between the end of one year and the beginning of the next.

The rectangle in panel A indicates the period shown in more detail in panel B (30 September 2019 - 08 March 2020). The vertical dashed line indicates the first autochthonous COVID-19 case diagnosis in Italy. The vertical dotted light lines indicate the 1 January of each year.

**Supplementary Figure S11** – Weekly number viral pneumonia hospitalizations (ICD-9-CM code 480): A) Observed, estimated and predicted value during the study period (from 29 September 2014 to 29 September 2019) in Lodi province, B) Observed and predicted value from the beginning of 2019 autumn season to the end of the study (from 30 September 2019 to 8 March 2020) in Lodi province. Hospital discharge record system, Italy, 29 September 2014 – 8 March 2020


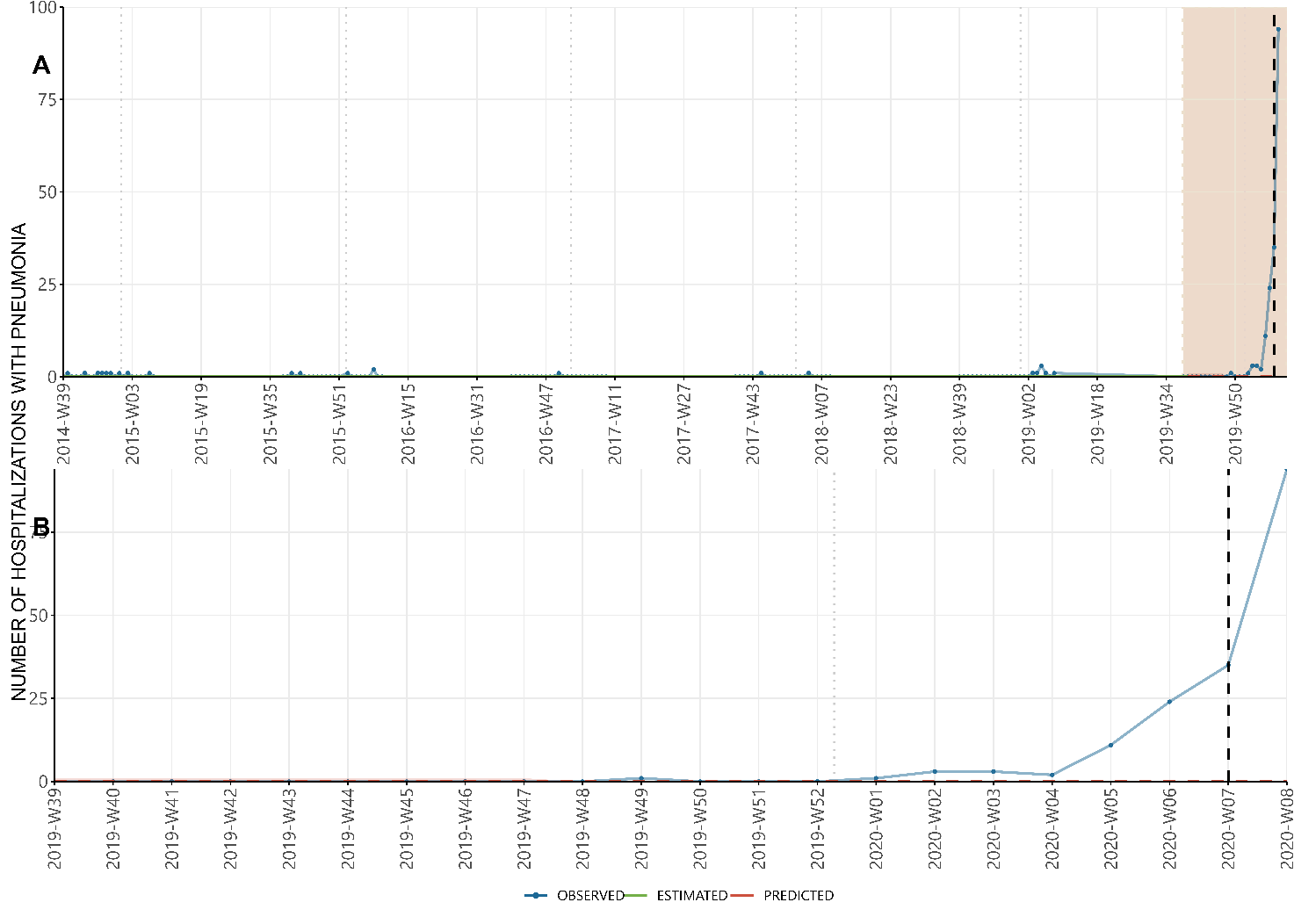


In order to facilitate graphical representation, the data has been formatted according to the isoweek convention. The analysis has correctly accounted for any weeks that fall between the end of one year and the beginning of the next.

The rectangle in panel A indicates the period shown in more detail in panel B (30 September 2019 - 08 March 2020). The vertical dashed line indicates the first autochthonous COVID-19 case diagnosis in Italy. The vertical dotted light lines indicate the 1 January of each year.

**Supplementary Figure S12** – Number of tested and negative specimens and percentage of negative specimens of ILI in Lombardy. National Health Service Sentinel System (InfluNet), Italy, seasons 2017-2018, 2018-2019 and 2019-2020


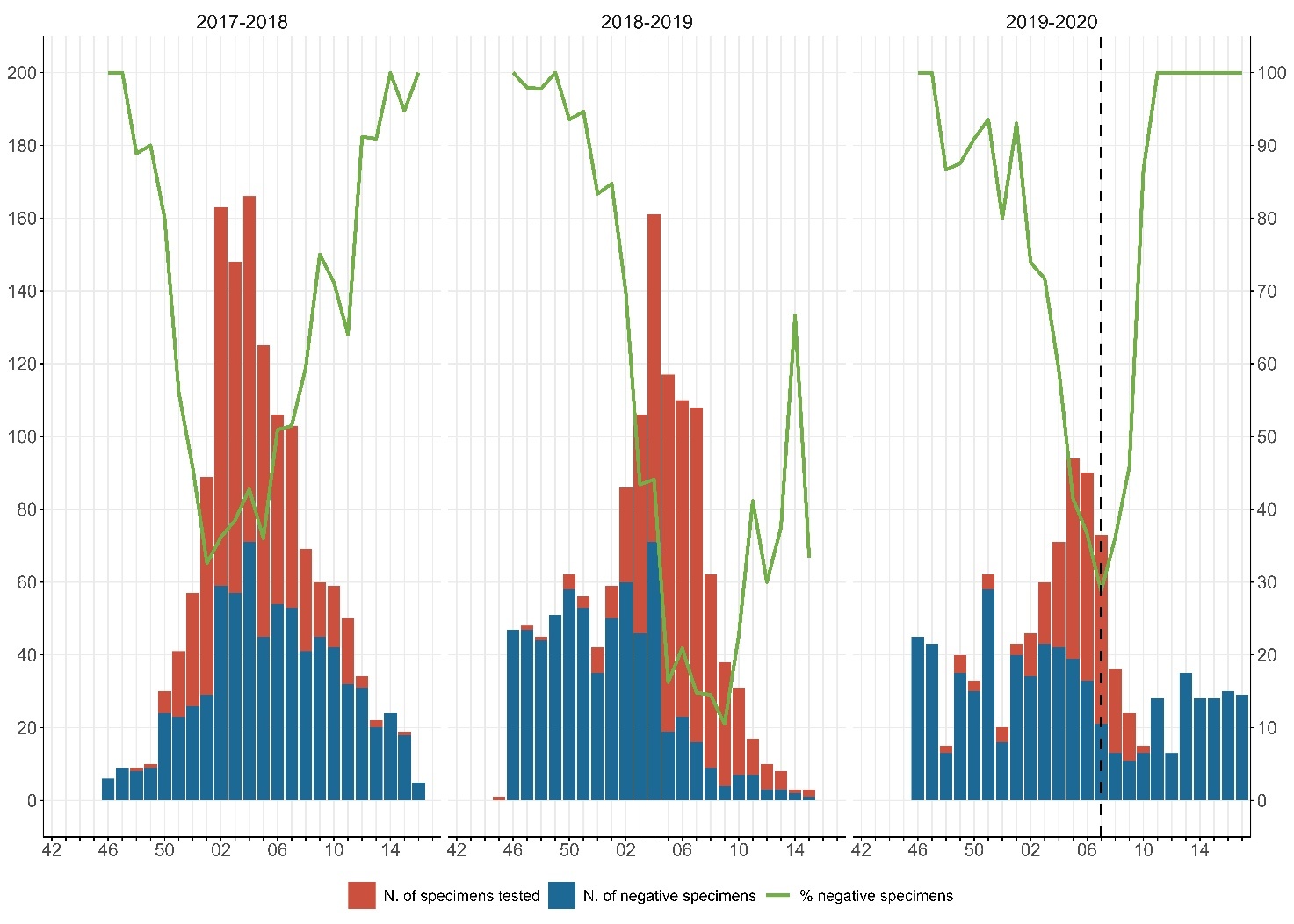


In order to facilitate graphical representation, the data has been formatted according to the isoweek convention. The analysis has correctly accounted for any weeks that fall between the end of one year and the beginning of the next.

The vertical dashed line indicates the first autochthonous COVID-19 case diagnosis in Italy.
