# Supplementary material for: Was severe SARS-CoV-2 substantially spreading in Northern Italy before its first detection in February 2020? An evaluation of pneumonia-associated hospitalization trends from September 2014 to February 2020
Source: Eur J Public Health. 2025 Aug 4;35(5):1050–7. doi: 10.1093/eurpub/ckaf137 (PMC12529277; doi:10.1093/eurpub/ckaf137)
Supplement: ckaf137_Supplementary_Data [file ckaf137_supplementary_data.zip › ckaf137_Supplementary_Data/suppl.docx]

**Supplementary file list**

- Supplementary Table S1
- Supplementary Figure S1-S6
  - Supplementary Figure S1
  - Supplementary Figure S2
  - Supplementary Figure S3
  - Supplementary Figure S4
  - Supplementary Figure S5
  - Supplementary Figure S6
- Supplementary Figure S7-S12
  - Supplementary Figure S7
  - Supplementary Figure S8
  - Supplementary Figure S9
  - Supplementary Figure S10
  - Supplementary Figure S11
  - Supplementary Figure S12

**Supplementary file titles/captions**

**Supplementary Table S1—**Selected ICD-9-CM codes (categories, subcategories, subclassifications) and their descriptions

* New ICD-9-CM code;

^#^ selected only the subcategories listed;

^o^ selected only the subclassifications listed.

**Supplementary Figure S1-S6**

**Supplementary Figure S1—**Weekly number of diagnosis and hospitalizations of SARS-CoV-2 reported to the Italian COVID-19 Integrated Surveillance System. Italy, 27 January 2020–8 March 2020.

In order to facilitate graphical representation, the data has been formatted according to the isoweek convention. The analysis has correctly accounted for any weeks that fall between the end of one year and the beginning of the next. The vertical dashed line indicates the first autochthonous COVID-19 case diagnosis in Italy.

**Supplementary Figure S2—**Weekly observed hospitalizations with pneumonia in **Lombardy provinces**, 30 September 2019—8 March 2020. Hospital discharge record system, Italy, 30 September 2019–8 March 2020

480 - viral pneumonia; 481 - pneumococcal pneumonia; 482 - other bacterial pneumonia; 483 - pneumonia due to other specified organisms; 484 - pneumonia in infectious diseases classified elsewhere; 485 - bronchopneumonia with unspecified organisms; 486 - pneumonia with unspecified organisms; 487.0 - influenza with pneumonia; 516.9 - unspecified alveolar and parietoalveolar pneumonopathy; 518.81 - acute respiratory failure; 518.82 - other pulmonary insufficiency not elsewhere classified; 770.0 - congenital pneumonia

In order to facilitate graphical representation, the data has been formatted according to the isoweek convention. The analysis has correctly accounted for any weeks that fall between the end of one year and the beginning of the next. The vertical dashed line indicates the first autochthonous COVID-19 case diagnosis in Italy. The vertical dotted light grey lines indicate the 1 January of each year.

**Supplementary Figure S3—**Weekly observed hospitalizations with a pneumonia ICD-9-CM code (any position): **A)** Observed value from 29 September 2014 to 08 March 2020 in **Bergamo province**, **B)** Same as A) focusing on the period from 30 September 2019 to 08 March 2020, **C)** Observed value from 29 September 2014 to 8 March 2020 in **Lodi province**, **D)** Same as C) focusing on the period from 30 September 2019 to 08 March 2020. Hospital discharge record system, Italy, 29 September 2014–8 March 2020

480 - viral pneumonia; 481 - pneumococcal pneumonia; 482 - other bacterial pneumonia; 483 - pneumonia due to other specified organisms; 484 - pneumonia in infectious diseases classified elsewhere; 485 - bronchopneumonia with unspecified organisms; 486 - pneumonia with unspecified organisms; 487.0 - influenza with pneumonia; 516.9 - unspecified alveolar and parietoalveolar pneumonopathy; 518.81 - acute respiratory failure; 518.82 - other pulmonary insufficiency not elsewhere classified; 770.0 - congenital pneumonia

In order to facilitate graphical representation, the data has been formatted according to the isoweek convention. The analysis has correctly accounted for any weeks that fall between the end of one year and the beginning of the next.

The light orange rectangle in panel A indicates the period shown in more detail in panel B (30 September 2019—08 March 2020). The vertical dashed line indicates the first autochthonous COVID-19 case diagnosis in Italy. The vertical dotted light grey lines indicate the 1 January of each year.

**Supplementary Figure S4—**Weekly observed hospitalizations with pneumonia in **Italian Regions/APs**, 1 October 2018—8 March 2020. Hospital discharge record system, Italy, 1 October 2018–8 March 2020

480 - viral pneumonia; 481 - pneumococcal pneumonia; 482 - other bacterial pneumonia; 483 - pneumonia due to other specified organisms; 484 - pneumonia in infectious diseases classified elsewhere; 485 - bronchopneumonia with unspecified organisms; 486 - pneumonia with unspecified organisms; 487.0 - influenza with pneumonia; 516.9 - unspecified alveolar and parietoalveolar pneumonopathy; 518.81 - acute respiratory failure; 518.82 - other pulmonary insufficiency not elsewhere classified; 770.0 - congenital pneumonia

In order to facilitate graphical representation, the data has been formatted according to the isoweek convention. The analysis has correctly accounted for any weeks that fall between the end of one year and the beginning of the next. The vertical dashed line indicates the first autochthonous COVID-19 case diagnosis in Italy. The vertical dotted light grey lines indicate the 1 January of each year.

**Supplementary Figure S5—**Weekly observed hospitalizations with a pneumonia ICD-9-CM code (any position): **A)** Observed value from 29 September 2014 to 08 March 2020 in **Central-Southern Italy**, **B)** Same as A) focusing on the period from 30 September 2019 to 08 March 2020. Hospital discharge record system, Italy, 29 September 2014–8 March 2020.

480 - viral pneumonia; 481 - pneumococcal pneumonia; 482 - other bacterial pneumonia; 483 - pneumonia due to other specified organisms; 484 - pneumonia in infectious diseases classified elsewhere; 485 - bronchopneumonia with unspecified organisms; 486 - pneumonia with unspecified organisms; 487.0 - influenza with pneumonia; 516.9 - unspecified alveolar and parietoalveolar pneumonopathy; 518.81 - acute respiratory failure; 518.82 - other pulmonary insufficiency not elsewhere classified; 770.0 - congenital pneumonia

In order to facilitate graphical representation, the data has been formatted according to the isoweek convention. The analysis has correctly accounted for any weeks that fall between the end of one year and the beginning of the next.

The light orange rectangle in panel A indicates the period shown in more detail in panel B (30 September 2019—08 March 2020). The vertical dashed line indicates the first autochthonous COVID-19 case diagnosis in Italy. The vertical dotted light grey lines indicate the 1 January of each year.

**Supplementary Figure S6—**Weekly observed hospitalizations with a pneumonia ICD-9-CM code (any position): **A)** Observed value from 29 September 2014 to 08 March 2020 in **Italy**, **B)** Same as A) focusing on the period from 30 September 2019 to 08 March 2020. Hospital discharge record system, Italy, 29 September 2014–8 March 2020.

480 - viral pneumonia; 481 - pneumococcal pneumonia; 482 - other bacterial pneumonia; 483 - pneumonia due to other specified organisms; 484 - pneumonia in infectious diseases classified elsewhere; 485 - bronchopneumonia with unspecified organisms; 486 - pneumonia with unspecified organisms; 487.0 - influenza with pneumonia; 516.9 - unspecified alveolar and parietoalveolar pneumonopathy; 518.81 - acute respiratory failure; 518.82 - other pulmonary insufficiency not elsewhere classified; 770.0 - congenital pneumonia

In order to facilitate graphical representation, the data has been formatted according to the isoweek convention. The analysis has correctly accounted for any weeks that fall between the end of one year and the beginning of the next.

The light orange rectangle in panel A indicates the period shown in more detail in panel B (30 September 2019—08 March 2020). The vertical dashed line indicates the first autochthonous COVID-19 case diagnosis in Italy. The vertical dotted light grey lines indicate the 1 January of each year.

**Supplementary Figure S7-S12**

**Supplementary Figure S7—**Weekly number pneumonia hospitalizations in **Italy: A)** Observed and estimated value before the 2019 autumn season (from 29 September 2014 to 8 March 2020), **B)** Observed and predicted value from the beginning of 2019 autumn season to the end of the study (from 30 September 2019 to 8 March 2020). Hospital discharge record system, Italy, 29 September 2014–8 March 2020

In order to facilitate graphical representation, the data has been formatted according to the isoweek convention. The analysis has correctly accounted for any weeks that fall between the end of one year and the beginning of the next.

The light orange rectangle in panel A indicates the period shown in more detail in panel B (30 September 2019—08 March 2020). The vertical dashed line indicates the first autochthonous COVID-19 case diagnosis in Italy. The vertical dotted light grey lines indicate the 1 January of each year.

**Supplementary Figure S8—**Weekly number pneumonia hospitalizations: **A)** Observed, estimated and predicted value during the study period (from 29 September 2014 to 29 September 2019) in **Bergamo province**, **B)** Observed and predicted value from the beginning of 2019 autumn season to the end of the study (from 30 September 2019 to 8 March 2020) in **Bergamo province**, **C)** Observed, estimated and predicted value during the study period (from 29 September 2014 to 29 September 2019) in **Lodi province**, **D)** Observed and predicted value from the beginning of 2019 autumn season to the end of the study (from 30 September 2019 to 8 March 2020) **in Lodi province**. Hospital discharge record system, Italy, 29 September 2014–8 March 2020

In order to facilitate graphical representation, the data has been formatted according to the isoweek convention. The analysis has correctly accounted for any weeks that fall between the end of one year and the beginning of the next.

The light orange rectangle in panel A indicates the period shown in more detail in panel B (30 September 2019—08 March 2020). The vertical dashed line indicates the first autochthonous COVID-19 case diagnosis in Italy. The vertical dotted light grey lines indicate the 1 January of each year.

**Supplementary Figure S9—**Weekly number of viral pneumonia hospitalizations (ICD-9-CM code 480): **A)** Observed, estimated and predicted value during the study period (from 29 September 2014 to 29 September 2019) in **Lombardy region**, **B)** Observed and predicted value from the beginning of 2019 autumn season to the end of the study (from 30 September 2019 to 8 March 2020) in **Lombardy** **region**. Hospital discharge record system, Italy, 29 September 2014–8 March 2020.

In order to facilitate graphical representation, the data has been formatted according to the isoweek convention. The analysis has correctly accounted for any weeks that fall between the end of one year and the beginning of the next.

The light orange rectangle in panel A indicates the period shown in more detail in panel B (30 September 2019—08 March 2020). The vertical dashed line indicates the first autochthonous COVID-19 case diagnosis in Italy. The vertical dotted light grey lines indicate the 1 January of each year.

**Supplementary Figure S10—**Weekly number viral pneumonia hospitalizations (ICD-9-CM code 480): **A)** Observed, estimated and predicted value during the study period (from 29 September 2014 to 29 September 2019) in **Bergamo province**, **B)** Observed and predicted value from the beginning of 2019 autumn season to the end of the study (from 30 September 2019 to 8 March 2020) in **Bergamo** **province**. Hospital discharge record system, Italy, 29 September 2014–8 March 2020.

In order to facilitate graphical representation, the data has been formatted according to the isoweek convention. The analysis has correctly accounted for any weeks that fall between the end of one year and the beginning of the next.

The light orange rectangle in panel A indicates the period shown in more detail in panel B (30 September 2019—08 March 2020). The vertical dashed line indicates the first autochthonous COVID-19 case diagnosis in Italy. The vertical dotted light grey lines indicate the 1 January of each year.

**Supplementary Figure S11—**Weekly number viral pneumonia hospitalizations (ICD-9-CM code 480): **A)** Observed, estimated and predicted value during the study period (from 29 September 2014 to 29 September 2019) in **Lodi province**, **B)** Observed and predicted value from the beginning of 2019 autumn season to the end of the study (from 30 September 2019 to 8 March 2020) in **Lodi** **province**. Hospital discharge record system, Italy, 29 September 2014–8 March 2020.

In order to facilitate graphical representation, the data has been formatted according to the isoweek convention. The analysis has correctly accounted for any weeks that fall between the end of one year and the beginning of the next.

The light orange rectangle in panel A indicates the period shown in more detail in panel B (30 September 2019—08 March 2020). The vertical dashed line indicates the first autochthonous COVID-19 case diagnosis in Italy. The vertical dotted light grey lines indicate the 1 January of each year.

**Supplementary Figure S12** - Number of tested and negative specimens and percentage of negative specimens of ILI in **Lombardy**. National Health Service Sentinel System (InfluNet), Italy, seasons 2017-2018, 2018-2019 and 2019-2020.

In order to facilitate graphical representation, the data has been formatted according to the isoweek convention. The analysis has correctly accounted for any weeks that fall between the end of one year and the beginning of the next.

The vertical dashed line indicates the first autochthonous COVID-19 case diagnosis in Italy.
